# Iatrogenic tracheobronchial rupture

**Published:** 2014-09-25

**Authors:** M Paraschiv

**Affiliations:** * General Surgery Clinic, "Bagdasar-Arseni" Emergency Hospital, Bucharest

**Keywords:** iatrogenic tracheal rupture, emergency intubation, non-operative treatment, surgical treatment

## Abstract

Abstract

Iatrogenic tracheobronchial ruptures most frequently occur during tracheal intubation, but they can also be produced during tracheobronchial endoscopy or thoracic surgery. The clinical presentation can be brutal, with respiratory failure, cervical emphysema, pneumothorax and hemoptysis. There are also less symptomatic presentations. The diagnosis is confirmed by bronchoscopy. The therapeutic approach can be differentiated, surgical or conservative, although the criteria are not universally accepted. This article aims to review the indications and therapeutic options.

## Introduction

Acute injuries of tracheobronchial tree are not very common but seriously life threatening. They can be produced by severe open or blunt cervical-thoracic trauma, or by iatrogenic causes such as tracheal intubation, tracheotomy, bronchoscopy or other per-oral instrumentations (tracheal or esophageal stent placement), operations (e.g., esophagectomy), etc. Iatrogenic causes are more frequent than traumatic etiology. The most common cause of iatrogenic tracheobronchial rupture is tracheal intubation.

Incidence 

 Post-intubation tracheobronchial rupture is a relatively rare complication, the data reviewed in literature showing a different incidence: less than 0.01% [**1**] or between 0.05 and 0.37% [**2-4**] of all oral intubation. For other iatrogenic tracheal injuries (during endoscopy, tracheotomy, and thoracic surgery), frequency is much lower.

Pathogenesis

 I. The trachea may be injured during endotracheal intubation, especially when it is performed in stressful, emergency conditions [**5-6**] by:

 - the stylets or tube tips placed excessively;

 - cuff over inflation that produces excessive stretching of the membranous wall, which may longitudinally tear [**7**];

 - endotracheal tube repositioning maneuvers without deflating the cuff [6,8,9].

 When producing tracheal injury during intubation, the following can also contribute:

 - the lack of experience of the anesthetist, with repeated intubation attempts and possibly insufficient anesthesia induction, that allows cough and movements of the head and neck of the patient during intubation [**6,7,9**];

 - double-lumen endotracheal tubes (commonly used in thoracic surgery), with the incidence of tracheal rupture between 0.05 and 0.35 % [**10**];

 - pushing the endotracheal tube too distal (may damage the carina or the primitive bronchi);

 - using a too thick endotracheal tube (more common situation for female patients).

 Other factors favoring post-intubation tracheal rupture are: congenital tracheal anomalies [**11-12**], female gender [**5**], height (less than 165 cm) [**5-6**], older age, poor biological condition, chronic obstructive pulmonary disease and other inflammatory lesions of the tracheobronchial tree [**11-12**], tracheomalacia [**6**], tracheal stenosis [**2-4,13**], disorders affecting the position of the trachea (mediastinal collections, lymph nodes, tumors) [**11-12**], chronic treatment with steroids (by consecutive weakening of the tissue) [**11-12**] .

 There are authors who defined the patient`s typology and the conditions most likely to produce the injury of the trachea at intubation: female, over 50 years old, who required a double lumen intubation and/or excessive pressures in the endotracheal cuff [**9**].

 II. During tracheotomy, the trachea can be injured by the stylet, the tip of the cannula and the needle or the dilator in the percutaneous version [**14**].

 III. A number of endoscopic procedures: rigid bronchoscopy, tracheal or esophageal stent placement, endoscopic tracheal treatments (laser therapy, diathermy), dilation of tracheal stenosis - may result in the rupture of the airway.

 IV. Direct tracheobronchial damage during open surgery (e.g. esophagectomy), favored by tumoral invasion, compression or inflammatory adhesion affecting the airway.

 Pathology: most post-intubation tracheal lesions occur in the posterior wall of the thoracic trachea.

 Cardillo et al. [**10**] classified the tracheal rupture by depth in the following levels:

 Level I - mucosal or sub-mucosal lesion without mediastinal emphysema and esophageal injury;

 Level II – complete tracheal lesion with mediastinal and subcutaneous emphysema, but without esophageal injury or mediastinitis;

 Level III A - complete lesion of the tracheal wall with a herniation of the mediastinal tissue and esophagus in the tracheal lumen, but without mediastinitis or esophageal rupture;

 Level III B - any rupture of the tracheal wall with esophageal injury or mediastinitis associated;

 Depending on the location, Leinung et al. [**15**] described three types of ruptures with therapeutic implications: type I can be repaired through right thoracotomy or trans-cervical approach, types II and III can be repaired only by thoracotomy.

- type I – lesion of the trachea to carina;

 - type II – lesion of the carina and main bronchi

 - type III - lobar and segmental bronchi

 Ruptures are usually linear affecting the membranous part of the thoracic trachea [**9-16**] most frequently (60-80%) in the distal third trachea [**5**].

 Clinical manifestations. The suspicion of iatrogenic tracheobronchial rupture is based on clinical signs and symptoms. Few patients are asymptomatic [**17**].

 - cervical- thoracic subcutaneous emphysema is the most common clinical manifestation (64.8%-80%) [**18-19**]; its degree varies depending on the location and magnitude of the airway lesion [**19**], and on the ventilation pressure. The cuff hyperinflation may temporary seal the lesion.

 - dyspnea and cyanosis;

 - hemoptysis (sometimes very discreet) [**20**];

 - pneumothorax - after the splitting of the mediastinal pleura. Positive pressure ventilation can cause hypertensive pneumothorax;

 - persistent air leak and lack of pulmonary re-expansion after pleural drainage

 - less common symptoms include chest pain (associated with slight cervical emphysema), hypotension (by decrease in cardiac filling due to mediastinal displacement);

 - acute mediastinitis is a rare complication following a rupture of the membranous portion of the trachea, in contrast with the esophageal perforation where mediastinitis is a constant discovery [**21**];

 The time between the injury and the clinical symptoms can largely vary – up to 72 hours [**22**]. A very late diagnosis is mentioned in literature, even at 240 hours [**18**].

 Radiological investigations: Standard cervical-thoracic radiograph may reveal early signs deep cervical and mediastinal emphysema (92% - 100%), especially on the profile radiography of the cervical spine [**23**]. Usually, pneumothorax is present as well. However, about 40% of the pneumothorax cases may be missed at a standard radiological exam and can be revealed only by CT scan [**23**].

**Fig. 2:**
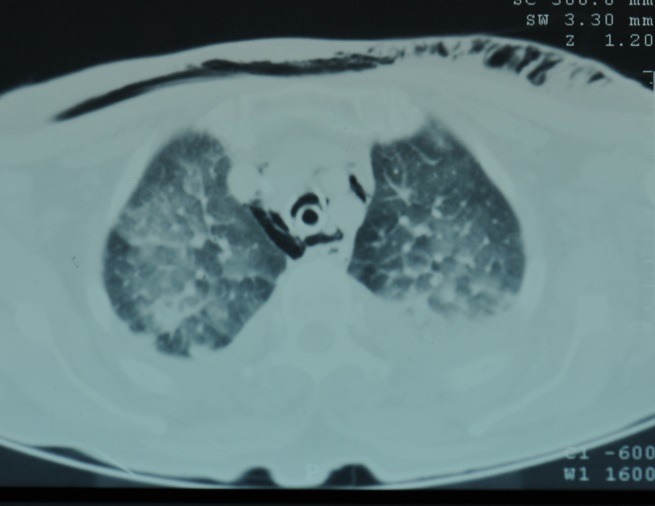
Gaseous thoracic syndrome after tracheal rupture (emergency intubation for broncho-pneumonia and bilateral pleuresia)

 CT scan shows the presence of extra-pulmonary air: cervical and mediastinal emphysema, pneumothorax, possibly pneumoperitoneum and pneumopericardium (**Fig. 2**).

 CT scan sensitivity in identifying the direct sign of rupture - discontinuity of the tracheal wall or deformation of its length - is about 71% [**23**]. CT multiplan reconstruction can diagnose tracheobronchial ruptures, providing positive results in 100% of the cases, without false negative results [**24**].

**Fig. 1:**
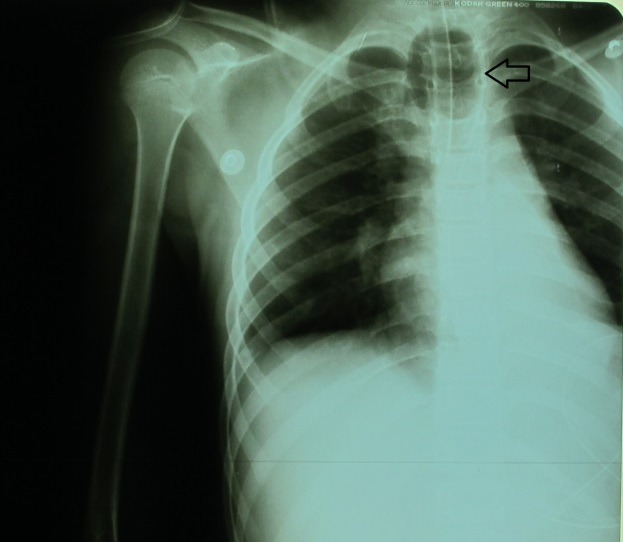
Chest x-ray showing the hyperinflated endotracheal cuff

**Fig. 3:**
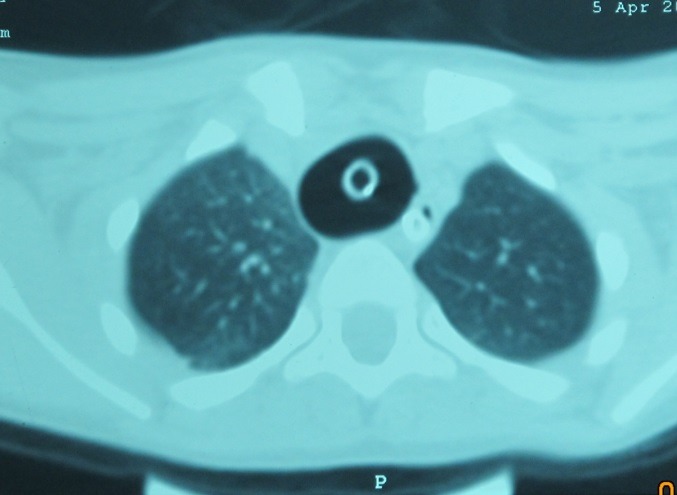
Tracheal rupture produced by overinflation of the endotracheal cuff. Minimal mediastinal emphysema

 In patients with a tracheal rupture who are intubated at the time of the CT examination, the shape and size of the endotracheal cuff can be suggestive for tracheal lesion. A cuff diameter greater than 2.8 cm on CT sections, spherical or ovoid shaped, possibly herniated through the tracheal wall (29% of the cases) [**23-25**] is an indirect sign of the tracheal tear (Fig. 1,3).

**Fig. 4:**
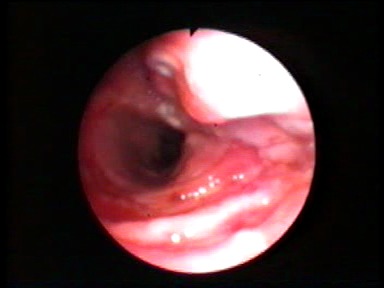
Endoscopic appearance of a large tracheal rupture with esophageal wall protrusion

 Bronchoscopy (**Fig. 4**) is the only investigation that can confirm the diagnosis of tracheobronchial rupture, directly visualizing the lesion, showing the exact location, extension (length and depth), and eventual herniation of the esophageal wall into the tracheal lumen. It may help in the planning of the therapeutic approach and can be used to reposition the tube or to re-intubate the patient.

 There can be exclusive endoscopic (intraluminal suture [**26**], application of fibrin gel) or endoscopic assisted (trans-cervical trans-tracheal approach) treatment options. Bronchoscopy is useful in monitoring the evolution of the conservative treatment, in reconsidering the non-operative attitude and for bronchial aspiration.

 The indication for bronchoscopy can be put early, before other investigations (radiography, CT scan), only on clinical suspicion, having the highest diagnostic accuracy [1-7,10,13,27,28]. Sometimes, bronchoscopy is performed in the operating room, on an endotracheal intubated patient, under general anesthesia, with a flexible bronchoscope. The endotracheal tube will be withdrawn over the bronchoscope up to the subglottic region and examination will be done in short periods of apnea.

Treatment 

 Until recently, the treatment of choice was represented by an emergency surgical repair of the lesion [2,5,6,29,30]. In recent years, conservative therapy has been indicated in some cases. In the only meta-analysis of the cases and series published in the Anglo-Saxon literature, Minambres and colleagues [**18**] noted the fact that a consensus on the therapeutic approach had not been reached.

 According to several authors [**1-19**], surgical treatment will be indicated in case of a trans-mural lacerations greater than 2 cm, especially those located para-carinal, with the esophageal wall prolapsed into the tracheal lumen. The extension of the gaseous syndrome (pneumomediastinum, subcutaneous emphysema), the presence of major air leak after pleural drainage or early signs of mediastinitis are also indications for surgical intervention.

 Patients with a rupture length of less than 2 cm or not involving the whole thickness of the tracheal wall, located in the cranial 2/3 of the trachea (allowing distal intubation) can be selected for the non-operative treatment, as well as patients with poor biological condition and high operatory risk [1-3,10,13,19]. Signs of mediastinitis or the worsening of the chest gaseous syndrome require surgery even in cases initially treated conservatively.

 Non-operative treatment 

 Conservative treatment options in mechanical ventilated patients are the following: 

 - endotracheal intubation (under bronchoscopy control) with the cuff positioned distally to the lesion;

 - pleural drainage - in case of pneumothorax; 

 - appropriate antibiotic [**18-19**], mucolytic and cough relieving therapy;

 - regular bronchoscopic aspirations [**19**]; 

 - careful monitoring for possible airway obstruction and mediastinal or pulmonary sepsis. 

 Even distal, para-carinal lesions, lend themselves for conservative treatment by using selective bilateral bronchial intubation [**26**].

 Surgical treatment: The presence of tracheal rupture raises anesthesia difficulties. Ventilation can be provided by oro-tracheal intubation with the endotracheal tube passed distal to the lesion, unilateral bronchoscopic guided ventilation, HFJV (high frequency jet ventilation), ventilation through the surgical field.

**Fig. 5:**
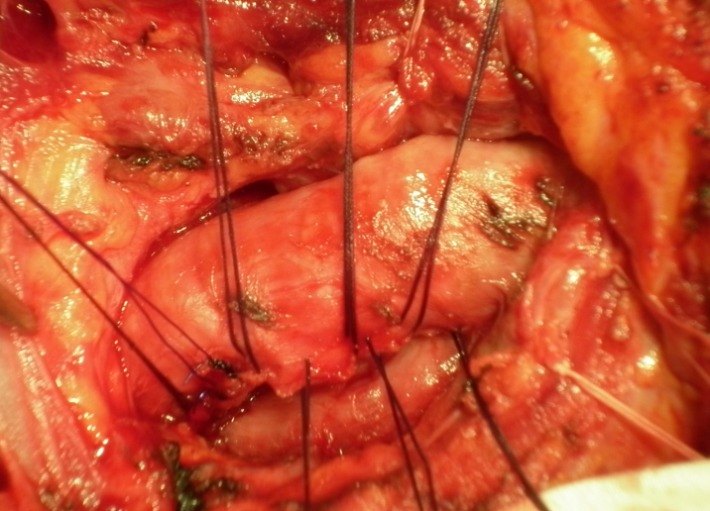
Cervical trachea rupture. Approach by transverse cervicotomy. Sutures (PDS 3-0) placed after minimal debridement of the rupture

 The choice of the surgical approach depends on the location and length of the rupture. Lesions of the larynx and proximal trachea are addressed by cervicotomy or cervical mediastinotomy (upper sternal division) (**Fig. 5**).

**Fig. 7:**
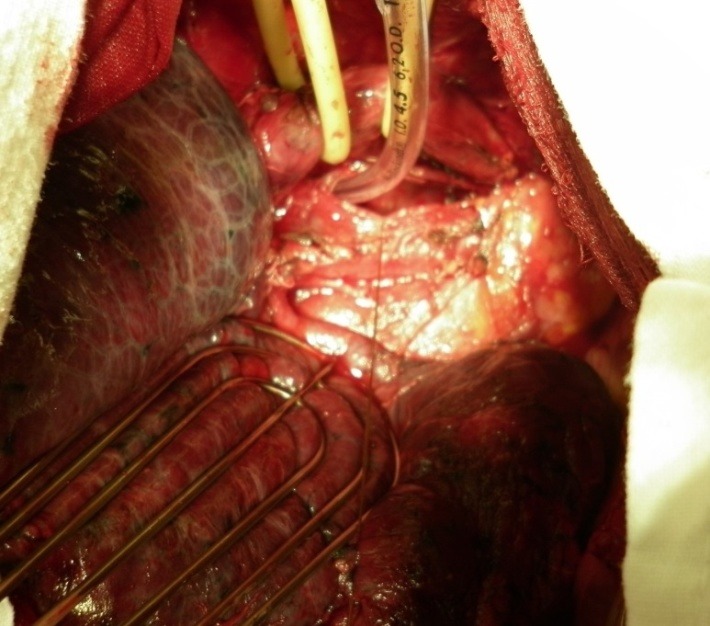
Intrathoracic tracheal rupture produced by overinflation of the cuff. Approach by right postero-lateral thoracotomy. Trachea exposed after dividing the azygos vein, intubated through the rupture. The esophagus is encircled and tractioned

**Fig. 8:**
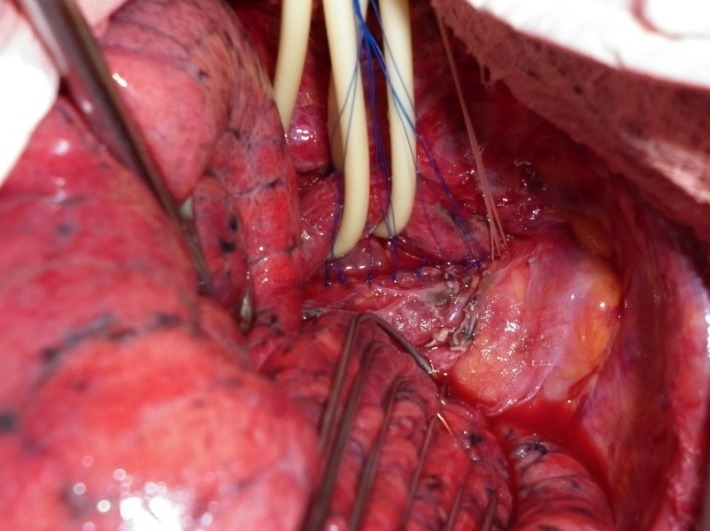
Tracheal suture (PDS 3-0)

Right posterolateral thoracotomy through the fourth or fifth intercostal space offers an excellent exposure to the distal trachea, the bifurcation of the trachea and both primitive bronchi [**19**]. Left thoracotomy will be preferred only for the isolated transversal ruptures of the left bronchus nearby the primitive lobar orifices [**4,7,10,19**]. Also, a double approach can be used: by cervicotomy and right thoracotomy (Fig. 7,8). Thoracoscopic minimally invasive techniques are mentioned [**31**].

**Fig. 6:**
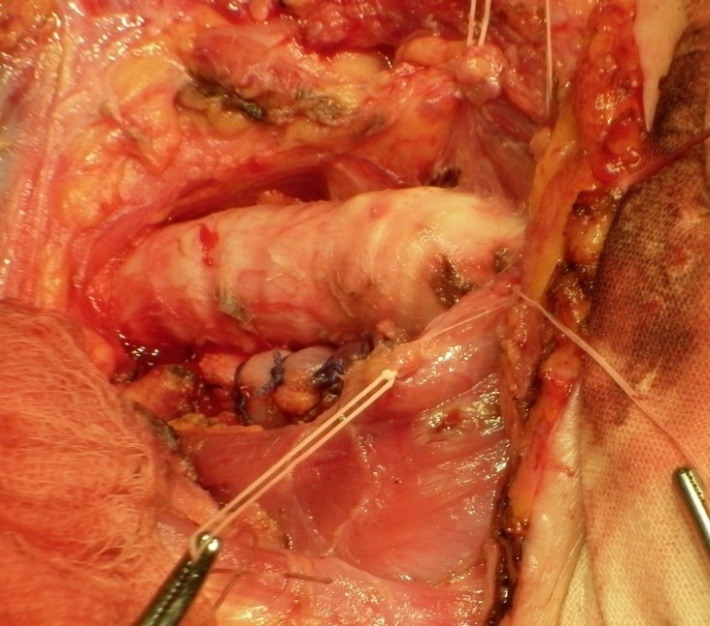
The tracheal suture is covered with sterno-hyoid muscle, rotated on the distal insertion. Note the traction sutures on the thyroid isthmus

 The surgery of the airway lesions must respect a few principles: minimal debridement of rupture edges, mucosa to mucosa repair, viable tissue plasty of the airway suture (muscle (**Fig. 6**), pericardium, pleura, mediastinal fat) [**32**]. The suturing material is absorbable; most authors recommend a running-suture, whenever possible. In patients with a late diagnosis, the presence of mediastinitis and inflammatory changes of the tracheobronchial wall, a simple suture is not allowed. In such cases, the use of muscle pedicled flap for covering the suture line and preventing the tracheal esophageal fistula formation is mandatory.

 After surgery, the patients will be extubated as soon as possible, preferably in the operating room [**19**] or within 24 hours [**33-34**].

 Postoperative complication, related exclusively to the therapeutic gesture, is the dehiscence of the tracheal suture with mediastinitis (in a mediastinal space enlarged by surgery) which often evolves fatally. The risk factors for tracheal suture failure are the following: mediastinitis before surgery, previous resection of the esophagus, delay in the diagnosis [**17**], postoperative mechanical ventilation with high pressures, forcing the anastomosis with the endotracheal cuff.

 A cervical trans-tracheal endoscopic assisted approach has been proposed for distal tracheal tears [**35**]. This technique is criticized because of the additional tracheal damage.

 Exclusive endoscopic technique requires a rigid bronchoscope, jet ventilation and a hybrid instrument - an "optical" needle-holder (12o Hopkins telescope and a needle-holder, bounded together in a fixed device) [**36**].

 In conclusion, iatrogenic lesions of the airway are not rare. The frequency of their occurrence is in relation to both objective conditions of instrumentation (emergency intubation, morphology of the patient) and experience and skills of the anesthetist, bronchoscopist, etc. In the historical evolution, the therapeutic management has evolved from surgical treatment for all cases to selective non-operative options, applicable to small tears without mediastinitis in patients breathing spontaneously, but also in serious cases with multiple associated illnesses in patients still ventilated. The selection criteria for the therapeutic options are still under debate.
